# Traumatic pseudoaneurysm of the peroneal artery following lower extremity fracture – A case report and review of the literature

**DOI:** 10.1016/j.tcr.2025.101148

**Published:** 2025-02-24

**Authors:** Christian Thomas Hübner, Philipp Vetter, Sandro-Michael Heining, Hans-Christoph Pape, Christian Hierholzer

**Affiliations:** Department of Traumatology, University Hospital Zurich, University of Zurich, Raemistrasse 100, 8091 Zurich, Switzerland

**Keywords:** Lower extremity fracture, Traumatic pseudoaneurysm, Peroneal artery, Intramedullary nailing, Tibia fracture, Embolization

## Abstract

This report documents a rare case of arterial aneurysm following a closed midshaft tibial and fibular fracture. Briefly, a 17-year-old female suffered a traumatic lower extremity fracture and was treated by intramedullary nailing of the tibia. After an uneventful postoperative course, she noted a painful and pulsating swelling of the lateral aspect of the lower extremity about 2 weeks post op. Diagnostic ultrasound confirmed formation of traumatic pseudoaneurysm of the peroneal artery adjacent to the fibular fracture. Subsequent endovascular treatment using coil-embolization was successful. Bony healing occurred in a timely fashion and the patient returned to pain free semi-professional sports activities.

## Introduction

High impact injuries in lower extremity fractures with fracture disruption of both the tibial and fibular bone are a rare entity, and are usually reported after crush injuries, or polytrauma. The characteristic soft tissue envelope leaving the anterior- medial aspect of the tibia exposed makes the lower leg prone to open fractures with concomitant soft tissue injuries of the anterior and medial aspect of the tibia. Lower extremity fractures present a high risk for the development of compartment syndrome and require careful monitoring of clinical signs of increased compartment pressure.

The anterior and lateral compartments of the lower leg protect the fibular bone and therefore open fibular fractures and vascular injuries of the fibular artery are rare complications. Vascular injuries may depend on fracture morphology. A displaced spiral fracture may directly cause vascular injury with complete or incomplete disruption of the vessel.

Traumatic vascular injuries may occur and may present in form of direct vascular perforation or disruption as well as vascular shear injuries.

Complete vascular disruption may cause significant ischemia distally to the site of injury with acute onset of typical and severe symptoms of ischemic pain.

Vascular shear injuries may result in endothelial intima injuries with secondary vascular occlusion often occurring several hours following traumatic injury.

The third form of vascular injury can be a formation of a pseudoaneurysm caused by partial injury of the arterial wall with consecutive extravasation of blood in the surrounding tissue. Dependent on the size of wall perforation, a pseudoaneurysm may rapidly increase in size over hours or slowly over days until it ruptures or causes compartment syndrome [1].

In this case presentation, we describe a young female patient suffering a traumatic tibial and fibular shaft fracture in the distal third who developed a traumatic pseudoaneurysm of the peroneal artery caused by dislocated spiral fracture of the fibula. The pseudoaneurysm presented with typical clinical symptoms of increased swelling and compartment pressure on day 13 after fixation, thus highlighting the importance of a diligent clinical follow up – the options for therapeutic interventions are addressed.

## Case report

The patient consented to utilization of blinded data concerning the case for publication.

A 17-year-old female student suffered a fall when performing trampoline jumping. A loud noise, associated with inability to get up was experienced by the patient, followed by inability of mobilization and weight bearing.

Diagnostic work up demonstrated a fracture close to the distal of the tibia and the fibula.

The tibial fracture presented as an oblique fracture and the fibular fracture at the same level as a spiral type with additional fracture spike.

After preoperative testing, surgical stabilization was performed using i.m. nailing of the tibia. A tibial nail with a diameter of 10 mm was inserted and 3 interlocking screws were inserted distally, two from the medial to the lateral cortex and a third screw from the anterior to posterior cortex.

Proximally, an interlocking bolt was inserted using the targeting device from the medial to the lateral cortex. Since the strictly transverse fracture pattern of the tibia offered sufficient axial support, a compression screw was inserted and interfragmentary compression was applied. It was decided not to separately stabilize the fibular fracture, as the fibular reduction was considered sufficient.

On postoperative day 1, the patient was mobilized out of bed and for the left leg weight bearing was allowed as tolerated. Soft tissues were unremarkable and no significant swelling or any clinical sign of compartment syndrome was noted.

Distal pulses of anterior and posterior tibial artery were repeatedly palpated. The perfusion of lower leg and foot was unremarkable, she was discharged home on postoperative day 5 on continuous prophylactic antithrombotic medication.

On postop day 13, the patient experienced pain sudden onset of symptoms and swelling of the left lower leg and presented at the ER. At the site of the fibular fracture, significant swelling and tenderness on palpation was noted. There was no sign of peripheral peroneal nerve compression or sensory or motor deficits.

Selective ultrasound studies demonstrated an enlarged pseudoaneurysm of the left fibular artery. Clinically, no signs of imminent or acute compartment syndrome were present. In addition, using ultrasound studies, a deep vein thrombosis (DVT) was ruled out.

**Fig. 1:**
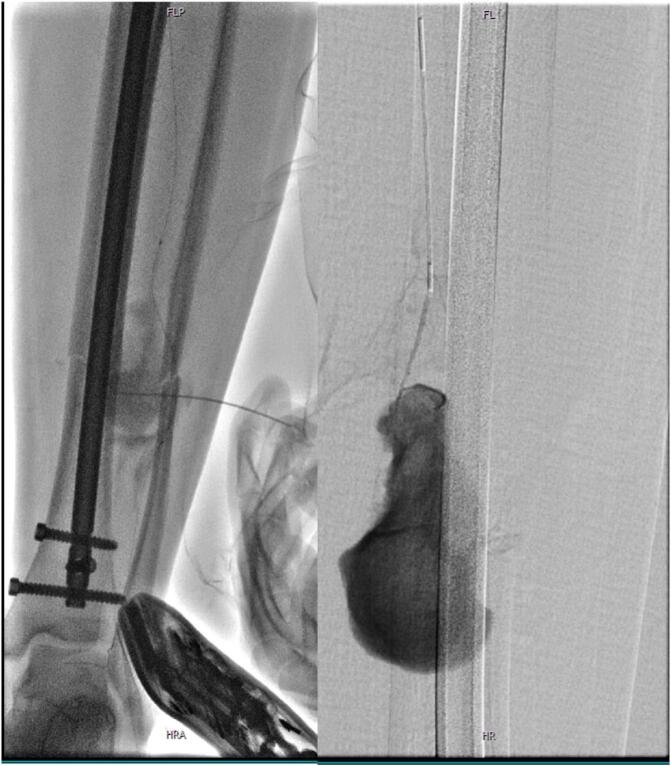
Angiography of traumatic pseudoaneurysm of the peroneal artery caused by oblique fracture morphology of the fibular fracture.

**Fig. 2:**
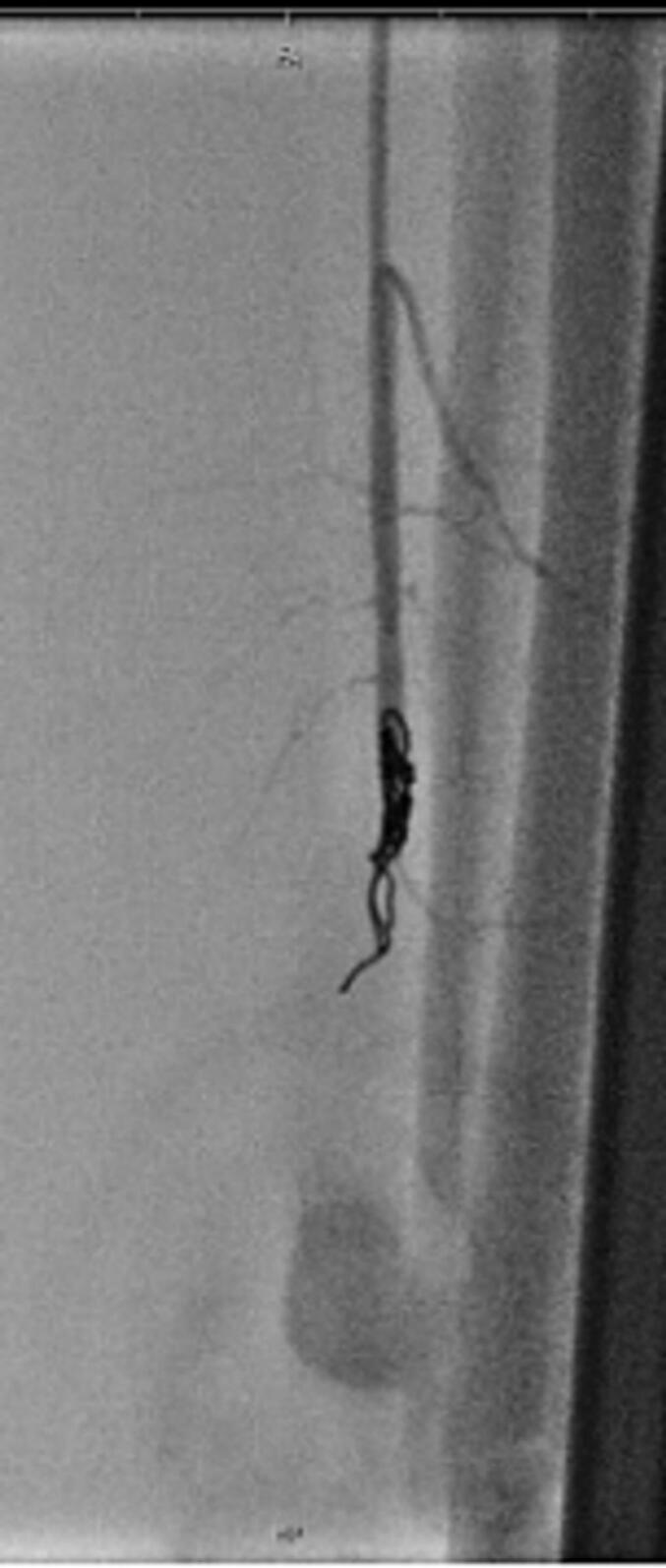
Successful embolization of the peroneal artery using intravascular coiling.

Therapeutically, the diagnosis of an “aneurysm spurium” was treated by selective embolization the left peroneal artery using PTA and coil embolization of the backdoor arteries (4× fig. 8 coil 2 × 5 mm) as well as feeding vessel (peroneal artery, 4× complex helical 6 × 6 mm, 1× interlock 6 × 10 mm) (Fig. 1, Fig. 2).

The consecutive course remained unremarkable.

**Fig. 3:**
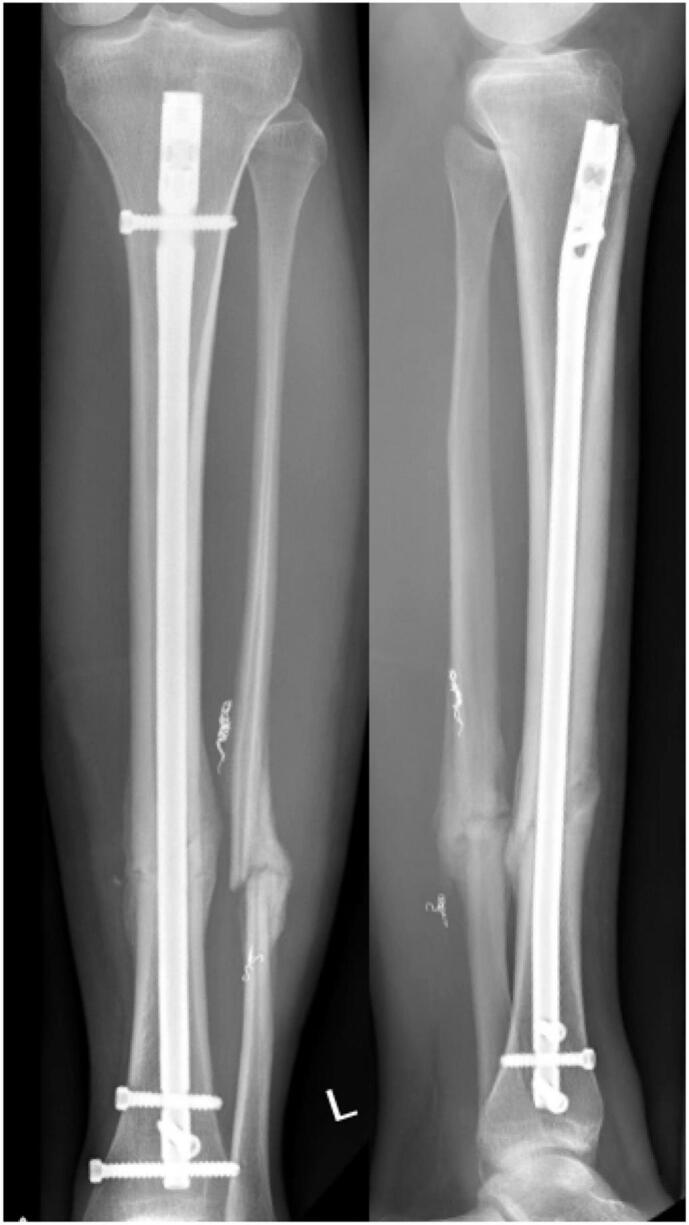
Postoperative X-ray control 6 weeks following surgery.

**Fig. 4:**
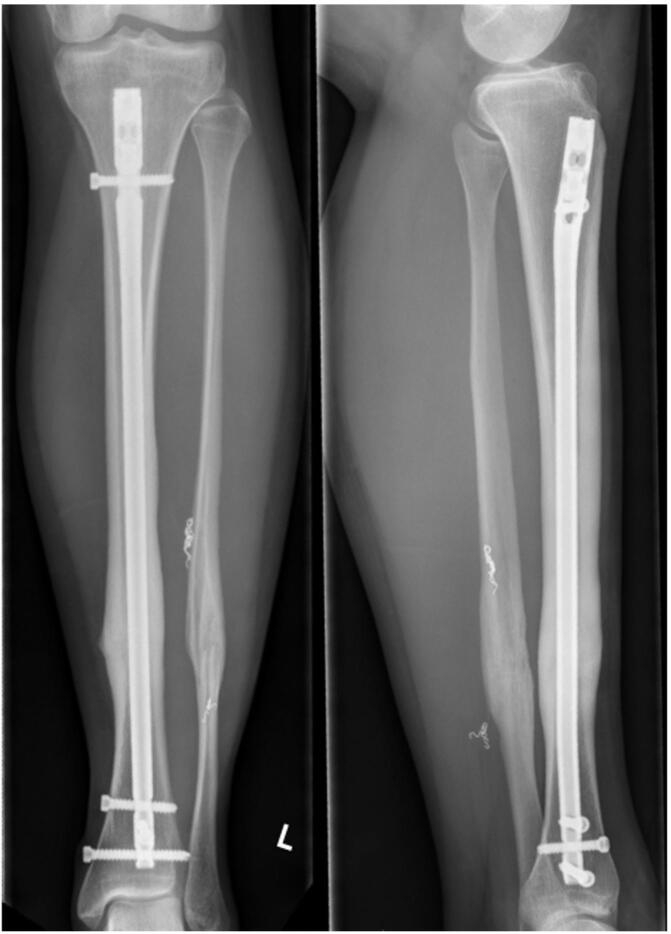
Follow up X-ray control one year following injury. Uneventful bony consolidation of the tibial and fibular fracture.

Follow up controls performed at regular intervals demonstrated timely and complete bony healing and no signs of perfusion deficits (Fig. 3, Fig. 4). The functional outcome was excellent and the young sportive patient was able to resume hockey and soccer team sports at a semiprofessional college level.

At the 2 year follow-up, the patient did not experience any long term signs of functional compartment syndrome or perfusion deficits when extensively performing extended sports activities.

## Discussion

The incidence of complications in tibia fractures is high compared to other anatomical locations. Beside a relevant reoperation rate of 12 to 44% [2], non-union rates of 3 % to 17 % are reported [3]. Furthermore infections are a major complication with about 1 % in closed tibia shaft fractures, and 25 to 30 % after complex open tibia shaft fractures [[3], [4], [5]]. In polytrauma the rate rises significantly up [6].

The incidence of peripheral false aneurysm independent of the type of injury is not clear overall. For the popliteal artery, the most common location of peripheral aneurysm, the prevalence in men over 65 years has been reported to be about 1 %. The second common location is in femoral artery [7]. Aneurysm of tibial and fibular artery are rarely described in literature.

Distally, pseudoaneurysm around the foot and ankle may also present in several locations predominately affecting the ATA and rarely the peroneal artery [8].

Inamdar et al. described iatrogenic injury of the ATA caused by insertion of a proximal interlocking screw several weeks after the procedure following an asymptomatic clinical interval. The associated risk with insertion of interlocking screws is discussed and the authors advise to apply proximal interlocking screws restrictively [9].

**Fig. 5:**
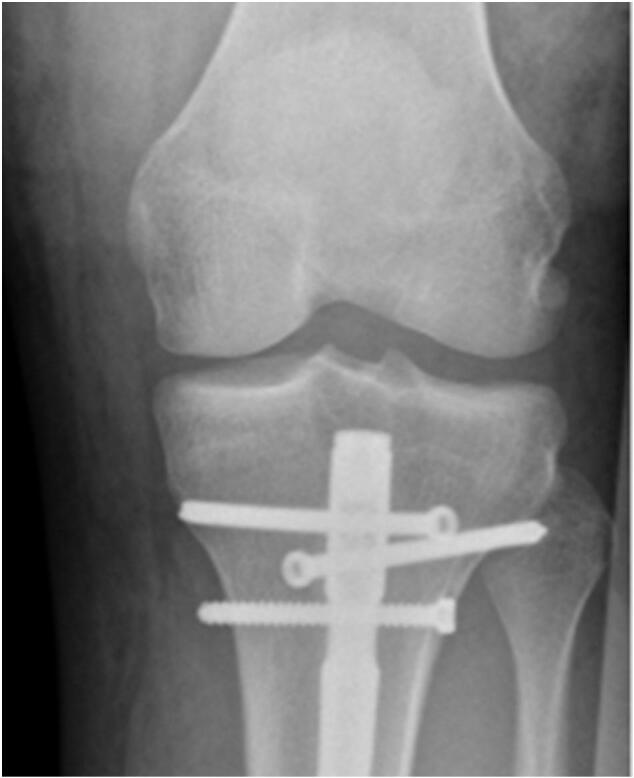
Proximal interlocking screws in unrelated case of i.m. tibial nailing with application of the interlocking screw in the dynamic position using high- risk lateral to medial insertion route.

In the study, our X-ray analysis of the films provided by the author revealed that insertion of the proximal interlocking screw was performed from the lateral to the medial cortex of the proximal tibia. This atypical position may be related to the risk of perforation of the anterior compartment with close proximity to the anterior tibial artery (Fig. 5).

According to the current recommendations, it is advised to insert 3 interlocking screws into the shorter fracture segment.

In distal shaft fractures of the tibia, the distal fragment bears two interlocking screws inserted from the medial to the lateral cortex and an additional a.p. locking screw.

However, singular cases of pseudoaneurysms were reported in literature where interlocking screws were inserted in favorable manner as well [10].

Placement of the distal a.p. locking screw may cause iatrogenic injury to the anterior neurovascular bundle. Frequently, the ATA demonstrates an expected anatomic course at the ankle.

### Issues with arterial variations

According to the knowledge taught in Anatomy books, the following course is supposed to be the rule: The ATA runs distally together with the deep peroneal nerve between the tendons of extensor hallucis longus and tibialis anterior.

In addition, Vasquez et al. and Fanter et al. demonstrated several variations of the anatomic course of the anterior tibial artery [11,12]. These included a more lateral course of the ATA crossing deep to the extensor digitorum longus and peroneus tertius to overlay the lateral malleolus before regaining its usual position in the foot, presence of an additional lateral branch, or in case of a hypoplastic ATA an enlarged perforating branch of the peroneal artery traversing the anterolateral tibia and assuming the course of the dorsalis pedis artery [11].

Therefore, application of the distal anterior locking screw requires cautious surgical preparation in order to avoid iatrogenic injuries to the anterior neurovascular bundle and extensor tendons. It is recommended to use a 2–3 cm skin incision and bluntly mobilize and retract the neurovascular bundle and extensor tendons until the anterior tibial cortex is directly visible. Using two Langenbeck retractors, drilling for locking screw insertion is only started if the corridor down to the tibial cortex is completely visible and liberated/cleared.

As shown in Table 1 pathogenesis of pseudoaneurysm of peroneal artery is often associated with operative treatment of fractures, and therefore, pseudoaneurysms are located in close proximity to foreign material (e.g. screw tips, Schanz-screws etc.).

**Table 1:** Peroneal pseudoaneurysms after lower leg fracture. Expanded and modified after Sankalp et al. [29]

Year	Authors	Reason for pseudoaneurysm	Formation through operative treatment likely	Treatment
1995	Tyllianakis et al. [15]	Tibia fracture and tibia nailing	Unlikely	Operative arterial repair
1999	Pai [19]	Ankle fracture and ORIF	Likely	Operative arterial suturing
2001	Montvilas et al. [20]	Open crural fracture	N.a.	N.a.
2002	Freiman et al. [21]	Closed fracture of the left proximal fibula, sharpnel blast	No	Embolization
2003	Kocakoc et al. [22]	Healed fibula fracture	Unlikely	Spontaneous healing
2003	Kurian et al. [23]	Ankle fracture and ORIF	Maybe	Embolization
2006	Henton et al. [24]	Calcaneus fracture and ORIF	Likely	Proximal ligation of the pseudoaneurysm
2013	Sala et al. [25]	Fracture and External fixator/Taylor Spatial Frame	Possible	Embolization
2016	Kosmidis et al. [13]	Weber B fracture and ORIF	Likely	Ultrasound-guided percutaneous thrombin injection
2019	Ileperuma et al. [26]	Tibia fracture and external fixator	Unclear	Proximal ligation of the pseudoaneurysm
2021	Cristiani-Winer et al. [27]	Weber fracture and ORIF	Likely	Embolization
2021	Pathinathan et al. [28]	Tibia and fibula fracture and external fixator	Likely	Proximal and distal ligation
2023	Sankalp et al. [29]	Non-union tibia-fracture, tibial nailing	Possible	Proximal and distal ligation
2024	This case	Tibia and fibula fracture and tibia nailing	Unlikely	Embolization

To the best of our knowledge, there are only three cases previously reported in literature, were formation of pseudoaneurysm seems to be provoked by the fracture itself and no correlation to operative treatment with screws or plates was visible.

### Additional factors in the pathogenesis of pseudoaneurysm

Interestingly, in the literature there are reports on pseudoaneurysm formation not only following tibia or fibular fractures of but also following ankle sprains. Predisposing factors may include underlying bony exostoses or simply shearing forces directed to the vessels, powerful enough to compromise the arterial wall [13]. Several referrals have been found in the published data of such injuries to the peroneal artery or to its perforating branches.

Additional comorbidities predispose patients to develop aneurysm formation including Marfan's disease, Ehlers-Danlos syndrome, Behcet's disease, type IV fibromuscular dysplasia, and osteogenesis imperfecta, whose collagen disorder inherently compromises the arterial wall integrity. In addition, infection combined with diabetes mellitus, malnutrition, and immunosuppression have been reported as predisposing factors [13].

### Posttraumatic and postoperative follow up

Following lower leg or ankle fractures careful and repeated assessment of the soft tissues and perfusion up to several days and weeks is required to detect traumatic injuries of the arterial blood supply.

ln presence of persistent and pronounced swelling of the lower leg and ankle joint, it is recommended to rule out DVT and arterial occlusion or pseudoaneurysm formation preferentially using ultrasound studies. Additional studies may include CT angiography, or MR angiogram [14].

Clinical phenotype oft DVT and Pseudoaneurysm can be overlapping, but precious diagnostic workup is absolutely necessary. Therapy of choice is completely different, since therapeutic anticoagulation in pseudoaneurysm can lead to severe complications [15].

As previous studies have also demonstrated, formation of pseudoaneurysm following sprain injuries of the ankle joint, these patients also require repeated and thorough clinical examinations during follow up visits in order to detect or rule out vascular injuries to the fibular artery. If clinical findings are not conclusive, ultrasound studies of the fibular artery are recommended.

### Therapy

Therapeutic options for pseudoaneurysm include minimally invasive endovascular treatment or open surgical interventions using suturing, grafting or ligation of the affected arterial vessel.

For endovascular treatment stent grafting or coil embolization can be utilized [16,17].

In the present case, coil embolization was selected because the diameter of the peroneal artery was too small for stent grafting and the blood flow distal to the pseudoaneurysm was disrupted.

It was decided to not apply open surgical intervention since no fasciotomy and open hematoma evacuation was required. This is in accordance with literature where endovascular treatment is more and more used beside open surgical repair (Table 1).

## Conclusion

In conclusion, pseudoaneurysms following operative treatment are rare complications. In our case it seems to be evocated by the fracture itself and not by the placement of interlocking bolts or other osteosynthesis material.

However, clinicians should be aware of this rare complication, as there are specific treatment options that differ from the more common complication of lower extremity swelling, a DVT.

Already in the pre-operative consent form patients and their family should be informed of the possibility of a pseudoaneurysm for medicolegal purposes. Postoperatively, the patient should be aware of any pulsating swelling occurs in the lower extremity and immediately consulate the operating surgeon or the emergency department. Careful follow up after operative treatment focusing the nerval and vascular status of patients is crucial. Morbidity and mortality conferences may be held to discuss this rare complication if it occurred in a hospital [18].

## Abbreviations

a.p.: anterior-posterior
ATA: arteria tibialis anterior
DVT: deep vein thrombosis
ER: emergency room
i.m.: intramedullary

## CRediT authorship contribution statement

**Christian Thomas Hübner:** Data curation, Investigation, Writing – original draft, Writing – review & editing. **Philipp Vetter:** Data curation, Writing – review & editing. **Sandro-Michael Heining:** Investigation, Writing – review & editing. **Hans-Christoph Pape:** Investigation, Validation, Writing – review & editing. **Christian Hierholzer:** Investigation, Supervision, Writing – review & editing.

## Funding statement

The authors received no financial support.

## Declaration of competing interest

The authors declare that they have no known competing financial interests or personal relationships that could have appeared to influence the work reported in this paper.
